# High-Temperature Adiabatic Calorimeter for Constant-Volume Heat Capacity Measurements of Compressed Gases and Liquids

**DOI:** 10.6028/jres.103.003

**Published:** 1998-02-01

**Authors:** Joseph W. Magee, Renee J. Deal, John C. Blanco

**Affiliations:** National Institute of Standards and Technology, Boulder, CO 80303; University of Colorado, Boulder, CO 80303

**Keywords:** adiabatic, calorimeter, gases, heat capacity, isochoric, liquids, measurements, water

## Abstract

A high-temperature adiabatic calorimeter has been developed to measure the constant-volume specific heat capacities (*c_V_*) of both gases and liquids, especially fluids of interest to emerging energy technologies. The chief design feature is its nearly identical twin bomb arrangement, which allows accurate measurement of energy differences without large corrections for energy losses due to thermal radiation fluxes. Operating conditions for the calorimeter cover a range of temperatures from 250 K to 700 K and at pressures up to 20 MPa. Performance tests were made with a sample of twice-distilled water. Heat capacities for water were measured from 300 K to 420 K at pressures to 20 MPa. The measured heat capacities differed from those calculated with an independently developed standard reference formulation with a root-mean-square fractional deviation of 0.48 %.

## 1. Introduction

Reliable thermal property data are required for efficient design in chemical engineering, as well as the enhancement of scientific understanding. The energy needed to increase the temperature of a kilogram of substance by 1 K (the specific heat capacity) is a quantity of considerable interest for many industrial applications. It also represents a fundamental measure of energy storage in the translational, rotational, and vibrational modes of a molecule and, as such, is useful in molecular theories.

An extensive array of techniques [1] have been developed to measure heat capacity. Of these techniques, the adiabatic method is generally accepted to give the most accurate results. The adiabatic method has been employed for heat capacity measurements by our research group for more than 35 years. Goodwin [2] developed a low-temperature adiabatic calorimeter, which was later modified by Magee [3] for automated measurement and control. It has been used to measure constant-volume heat capacities for many fluids at temperatures from 20 K to 345 K, and at pressures to 35 MPa. In the planning stages of this work, we recognized a need to augment our existing measurement capabilities by developing a new calorimeter which would extend the upper temperature limit to 700 K, without a compromise in accuracy.

In the adiabatic method, heat exchange between the calorimeter and its environment is eliminated as long as a temperature gradient does not exist. However, in actual practice the maintenance of a zero temperature gradient is an ideal situation which cannot be realized. Thus, even the most carefully conducted calorimetric experiment results in some heat loss. This heat loss can be minimized by automatic adjustment of the temperature of the surrounding jacket to follow that of the calorimeter.

Since in practice we are only able to minimize heat losses, the primary problem becomes how to accurately evaluate the correction for heat leakage. Fortunately, the magnitude of the heat-loss term is much less important than the accuracy with which it can be determined. Since an accurate calculation of heat leakage is difficult and often not possible, it would be advantageous to develop a technique which eliminates the need to make such a correction altogether. Early attempts to accomplish this goal employed twin calorimeters. Twin calorimeters were used by Joule [4] in the mid-nineteenth century and later by Pfaundler [5]. Such twin devices have in common two calorimeters as nearly identical in construction as possible, supported in nearly identical surroundings. In the present work, we combine the features of twin calorimeters with the adiabatic method, with the goal of realizing virtually complete elimination of the heat-loss correction.

## 2. Experimental Apparatus

**Fig. 1:**
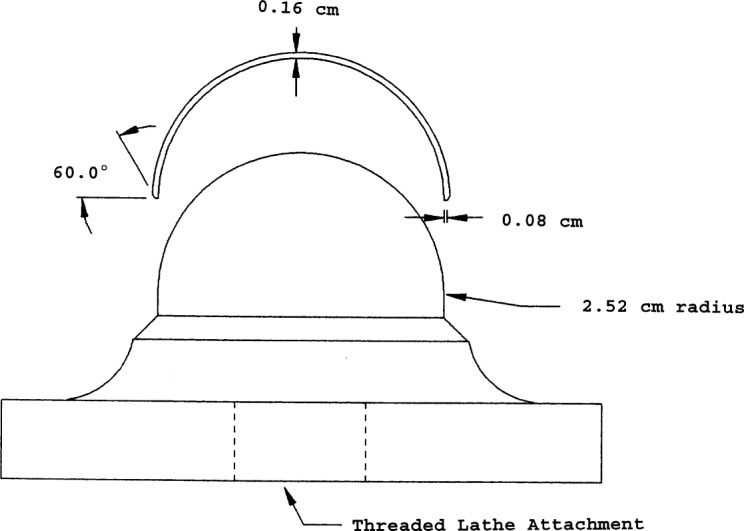
Details of Inconel hemispheres.

Almost identical spherical bombs of 70 cm^3^ capacity were designed to have a burst pressure in excess of 100 MPa at 700 K. The bombs were fabricated from Inconel 718 because of its high strength and excellent corrosion resistance. It is a weldable, machinable nickel-chromium alloy having exceptionally high tensile strength at elevated temperatures. Its tensile strength is more than twice that of Type-316 stainless steel at 700 K. A pair of these bombs was made from Inconel 718 steel sheet by spinning a hardened steel tool in a lathe to produce hemispheres which were subsequently welded together. As shown in Fig. 1, each hemisphere was machined with a face which is flat within close tolerances, and has a 608 beveled edge to permit better access by the welding tool when two halves are fused together. After the hemispheres were fused, a 0.16 cm diameter filler rod of the same composition was melted into the groove. After the excess filler material was removed by grinding, the surface was polished to give a smooth appearance to the sphere. A 0.64 cm OD Inconel 718 tube was then welded into a hole of the same size in the top of the sphere. The assembly was then annealed while it underwent an argon purge at 1200 K to remove any heat-induced stress. Each of the spheres was then radiographed to reveal flaws in the weldments, if any. Radiographs of the spheres were made at a wide angle to the weldment. This angled view would reveal any areas which were only partially fused together. Such flaws would have appeared as darkened areas. No flaws were detected. When it was established that the metal was fused through the entire wall, the cells were tested under high vacuum for any leaks. Then they were hydrostatically pressure-tested to 1.75 times the maximum operating pressure (to 35 MPa) to establish their strength. Wall stress calculations indicate that the upper pressure limit at 700 K, when the yield strength is exceeded, is approximately 110 MPa.

Selecting the heater wire and attaching this wire to the spheres proved to be most vexing problems. Most high-temperature wire insulations, such as polyimide and polytetrafluoroethylene, break down at temperatures greater than about 500 K. Epoxy adhesives also degrade at similar temperatures. An objection to silicone rubber is its propensity to outgas in vacuum. The choice for this work was a metal-sheathed heater which could be brazed onto the surface. An Inconel-sheathed, magnesium oxide insulated, nickel-chromium heater was brazed to the surface with a hard silver alloy that melted at about 900 K. A light steel jig was designed and built to hold the wires in place during brazing.

**Fig. 2:**
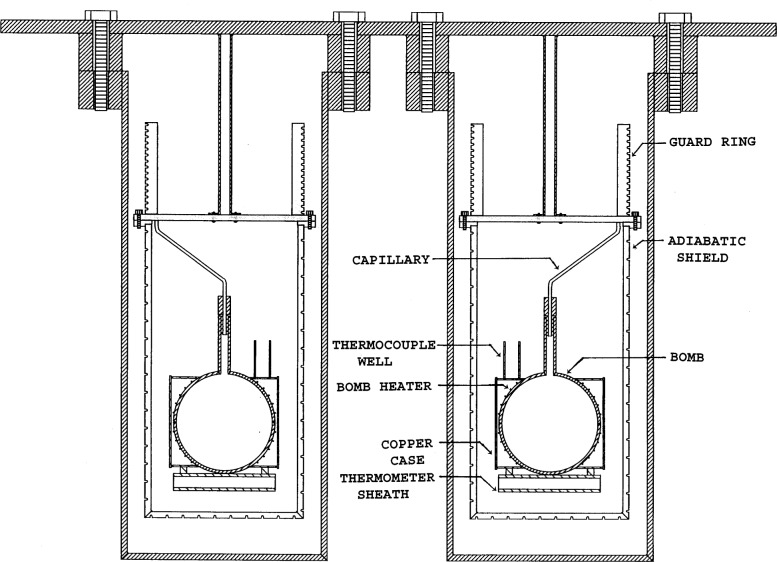
Schematic drawing of twin calorimeter.

As shown in Fig. 2, a light copper case was brazed to the sphere, covering two-thirds of its surface. It has two purposes. Chiefly, it intercepts stray heater radiation and conducts it back to the surface, thereby eliminating heat losses. Also, it serves as an anchor for the thermocouple well and platinum resistance thermometer (PRT) sheath which are brazed to it.

A capillary was attached to each sphere to fill and empty the vessels from outside the heated zone of the apparatus. A 0.05 cm inner diameter capillary was welded into a plug, which was in turn welded to the top of each 0.64 cm OD tube. Silver-alloy brazing could not be used to bond the parts of each sphere because of a known tendency of silver to catalyze decomposition of some hydrochlorofluorocarbon sample fluids at temperatures above 400 K. Silver and other brazing alloy components, notably copper, are also susceptible to corrosion with substances such as ammonia. The integrity of the weld seals was verified with a helium leak detector.

Each completed spherical bomb was jacketed in an adiabatic shield coupled to a guard ring, all constructed of Type-6061 aluminum, which was selected for its machinability and high thermal conductivity. The cylindrical surfaces of the guard ring and the sides and bottom of the shield were machined with spiral grooves to accept heaters which were pressed into them. The entire calorimeter assembly, shown in Fig. 2, has been placed inside a high-temperature forced-air convection furnace, specially designed for this application. A differential thermocouple provides a continuous reading of the temperature difference between the sample and the reference cells. A three-junction differential thermopile provides readings of the temperature difference between each bomb and its associated adiabatic shield. Type-K thermocouples encased in magnesium oxide insulation and sealed in a steel sheath were selected for this duty. The measuring ends of the three thermocouples were brazed to the inside surface of the shield with the active portion inserted in three holes which were separated by a 1208 angle from each other and located at an elevation near the center of the bomb. The three reference ends were bound together and placed in the thermocouple well which contained thermally conductive grease to a depth of 1 cm. A differential Type-K thermocouple provides readings of the temperature difference between the guard ring and the bomb. It was attached at each end in the same way as the shield thermocouples. A platinum resistance thermometer (PRT), having a calibration traceable to the National Institute of Standards and Technology, was coated with thermal grease and then inserted into the well brazed to the bottom of each bomb. These two thermometers provide measurements of the absolute temperature of each sphere with a high precision and accuracy. An oscillating quartz crystal pressure transducer, attached to the charging manifold, provides measurements of pressure. These pressures are nearly as accurate as the NIST-traceable piston gauges used to calibrate them. Each of the eight heaters (bomb, shield side, shield bottom, and guard for each cell) in the calorimeter is driven by independently controlled direct current power supplies. All of the instrumentation is connected to a microcomputer through an IEEE-488 standard interface bus. The computer executes FORTRAN code which has both temperature control and data acquisition functions. Except for sample charging, the new calorimeter is completely automated.

## 3. Principle of Operation

The basic principles of the heat capacity experiment are deceptively simple. For a single bomb, we measure the temperature rise (Δ*T*) when a measured quantity of heat energy (*Q*) is supplied to the calorimeter, which contains a mass (*m*) of substance. Subtracting the heat energy needed to heat the empty bomb (*Q*_0_) also widely known as *heat equivalent*, we calculate the sample heat capacity,

$$c_{V}=\left(Q-Q_{0}\right)/m\Delta T.$$ (1)

For a twin calorimeter, we simply replace values for *Q* with the differential quantity, Δ*Q*. It is defined as the heat energy supplied to the sample bomb minus that to the reference. The value of Δ*Q* accounts for the heat absorbed by the sample fluid. When we subtract the energy difference between the empty sample bomb and the reference, Δ*Q*_0_, we calculate the specific heat capacity at constant volume,

$$c_{V}=\left(\Delta Q-\Delta Q_{0}\right)/m\Delta T.$$ (2)

Equation (2) is the apparatus working equation. Experience with a single-cell calorimeter, has shown that the quantity *Q*_0_ in Eq. (1) is as much as 95 % of the measured heat energy *Q*. As a result, the relative expanded uncertainty (coverage factor *k* = 2 and thus a 2-standard-deviation estimate) propagated to *c_V_* from the total uncertainty in evaluating *Q*_0_ (≈ 0.02 %) may be up to 0.4 %. This shortcoming of the single-bomb calorimeter leads to an estimated expanded uncertainty for the heat capacity of 0.5 % for liquid and 2 % for gas samples [3]. A twin-bomb calorimeter overcomes this problem. The quantity Δ*Q*_0_ in Eq. (2) is small by design and, in practice, is nearly 0. The result of this technique is to make the uncertainty propagated from the heat equivalent measurements a negligible effect. Thus, we have improved the accuracy of the measurements, allowing us to achieve our goal of a relative expanded uncertainty of 0.4 % for the measured specific heat capacities at constant volume.

## 4. Performance Tests

The empty calorimeter function was determined from heating the completely evacuated bombs. Heating runs over the temperature range 300 K to 400 K were repeated until we were confident in the precision of the results. The data were fit by the function,

$$\Delta Q_{0}=2.13785\;\;\times \;\;10^{-2}J\cdot K^{-1}\;T-5.86322\;J.$$ (3)

Over 300 individual measurements agreed with Eq. (3) within a maximum deviation of ± 0.5 J. This equation is linear in temperature and ranges from 0.5 J to 3 J at temperatures from 300 K to 400 K.

A twice-distilled sample of water was prepared for a performance test of the new calorimeter. The water was charged into the calorimeter with a high-pressure syringe pump. Excess sample was slowly removed until the target pressure was reached. Measurements were initiated by applying a constant 3 V to the reference bomb heater. The low power (0.075 W) dissipated in this 120 Ω heater resulted in a temperature ramp rate of 0.04 K·min^−1^. The computer code quickly adjusted the sample bomb voltage to keep the bombs in thermal equilibrium within ± 5 × 10^−3^ K. When at equilibrium, the computer recorded the raw data needed for the heat capacity calculations. Heating cycles continued until the fluid pressure reached 20 MPa, the maximum pressure. Then the instruments were automatically reset to cool to the initial temperature of the run, and the run was repeated. After measurements were completed for a given isochore, a small amount of fluid was discharged into a light stainless-steel cylinder for weighing. After the last run, the remaining sample fluid was weighed.

**Fig. 3:**
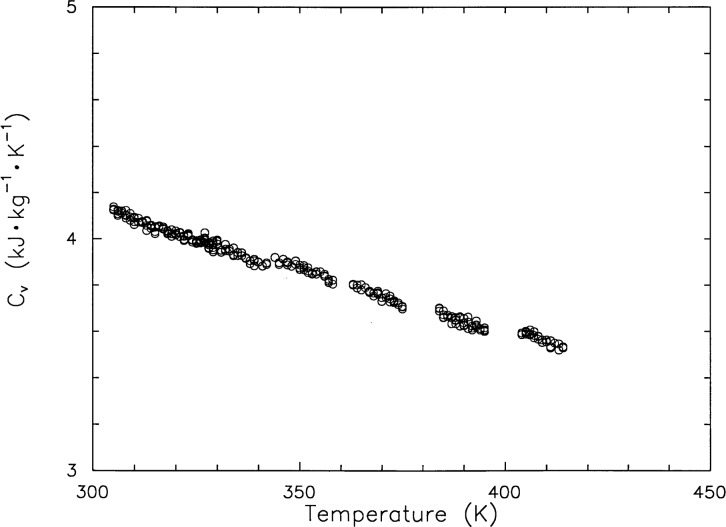
Measured specific heat capacity at constant volume *c_V_* for H_2_O.

**Fig. 4:**
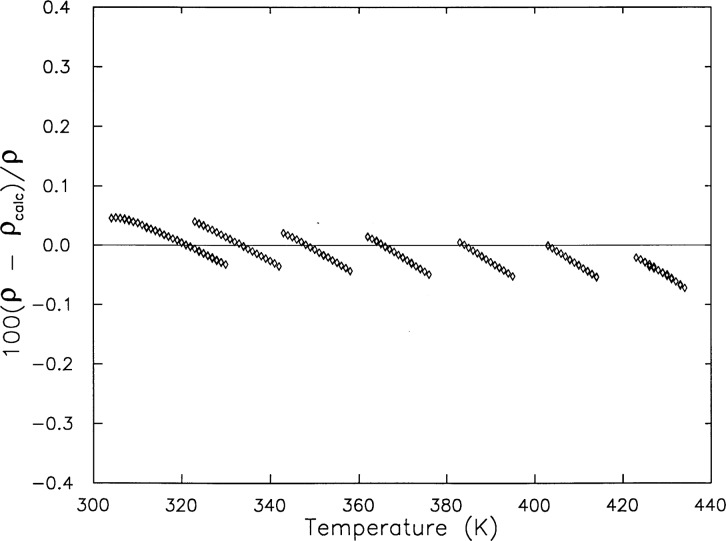
Deviations of H_2_O densities as determined in this work (◊) from densities calculated with the equation of state of Pruβ and Wagner [7] (baseline at deviation = 0.0).

Table 1 presents measurements of specific heat capacity at constant volume for water. The measurements are depicted in Fig. 3. To facilitate the comparisons with calculations with predictive models, this table gives more significant figures than would be normally justified. Experimental temperatures (*T*, ITS-90), pressures (*p*), and masses (*m*) are presented alongside the heat capacity (*c_V_*) data. These state values were used to establish the volume of the sample bomb. This quantity was calculated from *m*/*V*_calc_(*T*, *p*), where *V*_calc_ is calculated with the equation of state in the NBS/NRC Steam Tables of Haar, Gallagher, and Kell [6]. The calculated volume (*V*_b_), as a function of both temperature (*T*) and pressure (*p*), was fitted to the equation,

$$V_{b}=\left[V_{r}+c_{1}\left(T-273.15\;K\right)\right]\left[1+c_{2}p\right],$$ (4)

where *V*_r_ = 69.464 cm^3^, *c*_1_ = 3.2 × 10^−3^ cm^3^·K^−1^, and *c*_2_ = 1.36 × 10^−4^ MPa^−1^. Figure 4 shows the deviations of experimental densities from those calculated with the recently adopted international standard formulation by Pruβ and Wagner [7]. Since these experimental densities were determined from the mass of water found in Table 1 and the bomb volume calculated with Eq. (4), this comparison is intended to test how well Eq. (4) represents the sample bomb volume as a function of *T* and *p*. Deviations from the equation of state of Ref. [7] were not greater than 0.1 % and gave a root-mean-square fractional deviation of 0.05 %. Since we have found that densities calculated with Ref. [6] differ by less than 0.006 % from those calculated with Ref. [7] in the range of *T* and *p* of this work, we could justify the use of either of these formulations for our comparisons.

**Fig. 5:**
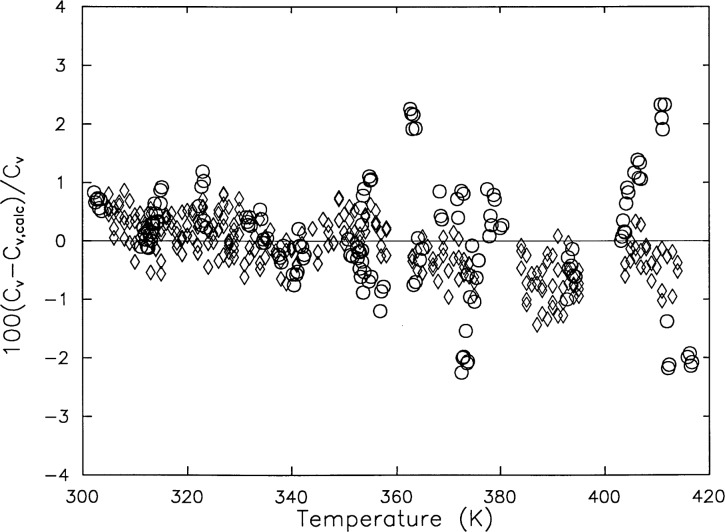
Deviation of measured H_2_O specific heat capacities at constant volume *c_V_* of this work (◊) and of Ref. [8] (○) from heat capacities calculated with the equation of state of Prub and Wagner [7] (baseline at deviation = 0.0).

**Fig. 6:**
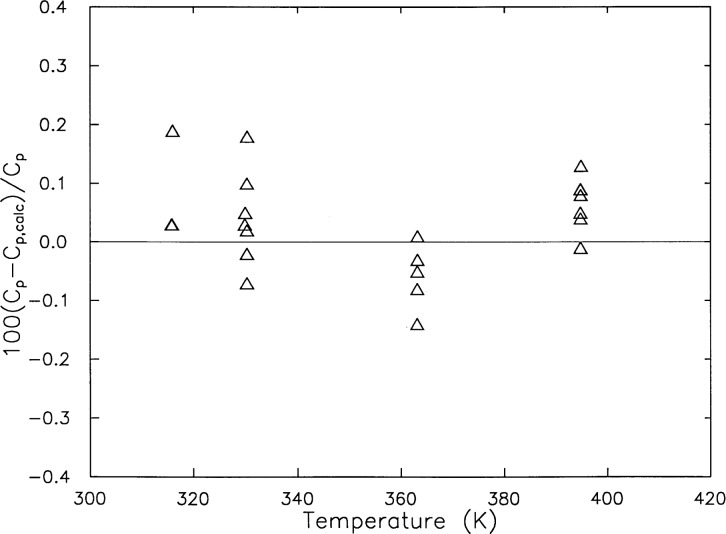
Deviation of measured H_2_O specific heat capacities at constant pressure *c_p_* of Ref. [9] (◊) from heat capacities calculated with the equation of state of Pruβ and Wagner [7] (baseline at deviation = 0.0).

Comparisons of the *c_V_* measurements were made with published values. Figures 5 and 6 shows comparisons with the calculations based on the Prub and Wagner formulation [7] at temperatures from 300 K to 420 K. The deviations of *c_V_* from this study shown in Fig. 5 did not exceed ± 1 % and gave a root-mean-square fractional deviation of 0.48 %. Figure 5 shows good agreement of this work with the published *c_V_* data of Amirkhanov et al. [8], which have an uncertainty of approximately 3 %. Figure 5 illustrates that deviations of the present data are distributed uniformly above and below the baseline representing the calculation of Pruβ and Wagner. The same comment applies to the published data of Ref. [8], except that they fall within a ± 3 % band. Since no other *c_V_* data were found in the temperature range of this study, we decided to make indirect comparisons with published specific heat capacity at constant pressure *c_p_* data from Sirota and Mal’tsev [9], which have an uncertainty of approximately 1 %. Figure 6 shows the deviations of the *c_p_* data from calculations made with the Pruβ and Wagner formulation. All deviations of the Ref. [9] data are within the claimed uncertainty of the published data, and fall in a ± 0.2 % band. Based on both direct and indirect comparisons, we conclude that the present results are in very good agreement with published *c_V_* and *c_p_* data.

## 5. Assessment of Uncertainties

Uncertainty in *c_V_* arises from several sources. Primarily, the accuracy of this method is limited by the uncertainty involved in the temperature rise measurement and the change-of-volume work adjustment [3]. In the following discussion, we use a definition for the expanded uncertainty which is two times the standard uncertainty (i.e., a coverage factor *k* = 2 and thus a 2-standard-deviation estimate).

Different sources of uncertainty, including calibration of the platinum resistance thermometer, radiation to or from the thermometer head, and drift of the ice point resistance, contribute to an expanded uncertainty of 3 × 10^−2^ K for the absolute temperature measurement. Uncertainty in the temperature rise measurement, however, also depends on the reproducibility of temperature measurements. The temperatures assigned to the beginning (*T*_1_) and to the end (*T*_2_) of a heating interval are determined from a linear fit of temperature with elapsed time, near the integer degree. The experimental ramp rate is approximately + 4 × 10^−2^ K·min^−1^. This procedure leads to an uncertainty of 5 × 10^−4^ K for the interpolated temperatures *T*_1_ and *T*_2_, leading to values of 7 × 10^−4^ K for the uncertainty of the temperature rise, Δ*T* = *T*_2_ − *T*_1_. For a typical experimental value of Δ*T* = 1 K, this corresponds to a relative uncertainty of 0.07 %.

The uncertainty of the change-of-volume work adjustment influences primarily the single-phase values since two-phase experiments are performed over a small pressure range. For water, the ratio of change-of-volume work to total applied heat is as large as 0.04 for the lowest density isochore. Estimated relative uncertainties of 2 % in the change-of-volume work are due to both the deviation of the calculated pressure derivatives and the uncertainty of the volume change. This leads to a relative uncertainty in *c_V_* of 0.08 %.

The energy applied to the calorimeter is the integral of the product of voltage and current from the initial to the final heating time. Voltage and current are measured 80 times during a heating interval of 1 K. The measurements of the electrical quantities have a relative uncertainty of 0.02 %. However, we must account for the effect of radiation heat losses or gains which occur when a spurious lag of the controller leads to a small temperature difference of about 10^−2^ K between bomb and radiation shield. Since heat transfer by radiation is proportional to *T*_1_^4^ − *T*_2_^4^ ≈ 4*T*^3^Δ*T*, we would expect radiation losses to substantially increase with the bomb temperature, and the losses may be different from the sample and from the reference bombs. Therefore, the uncertainty in the applied heat Δ*Q* is evaluated to be 0.5 J·K^−1^.

The energy difference Δ*Q*_0_ applied to the empty calorimeter has been measured in repeated experiments and fitted to a function of temperature. Its uncertainty is less than 0.5 J·K^−1^. Its influence on the uncertainty of the heat capacity is relatively small, because the ratio of the total heat Δ*Q* to the heat applied to the empty calorimeter Δ*Q*_0_ ranges from 100 to 600. The mass of each sample was determined within 0.01 % by differential weighings before and after trapping the sample. The density calculated from this mass and the bomb volume has a relative uncertainty of approximately 0.2 %. For pressures, the uncertainty of the gauge of 7 kPa is added to the cross term for the pressure derivative in the change-of-volume work adjustment. However, neither the uncertainty of *p* nor *ρ* contributes appreciably to the combined uncertainty for the measured heat capacity. The relative uncertainty of *c_V_* is determined to be 0.3 %, by combining the various sources of experimental uncertainty using a root-sum-of-squares formula.

## Figures and Tables

**Table 1:** Measurements of specific heat capacity at constant volume *c_V_* for H_2_O: *T*, temperature (ITS-90); *p*, pressure; *m*, sample mass; *c_V_*_,exp_, experimental heat capacity; *c_V_*,_calc_, heat capacity calculated with the equation of state of Ref. [7]; *D* = 100 (*c_V_*_,exp_ − *c_V_*,_calc_)/*c_V_*_,exp_

*T*_1_	*T*_2_	*p*_1_	*p*_2_	Δ*p*	*m*	*c_V_*_,exp_	_*cV*,calc_	*D*
(K)	(K)	(MPa)	(MPa)	(MPa)	(g)	(kJ·kg^−1^·K^−1^)		(%)
304.0	305.0	1.0100	1.4560	0.4460	69.3106	4.1288	4.1052	0.571
305.0	306.0	1.4560	1.9460	0.4900	69.3106	4.1232	4.0995	0.575
306.0	307.0	1.9460	2.4670	0.5210	69.3106	4.1115	4.0936	0.434
307.0	308.0	2.4670	3.0140	0.5470	69.3106	4.1233	4.0877	0.863
308.0	309.0	3.0140	3.5830	0.5690	69.3106	4.1099	4.0818	0.685
309.0	310.0	3.5830	4.1680	0.5850	69.3106	4.0939	4.0757	0.443
310.0	311.0	4.1680	4.7870	0.6190	69.3106	4.0889	4.0696	0.471
311.0	312.0	4.7870	5.4360	0.6490	69.3106	4.0695	4.0634	0.149
312.0	313.0	5.4360	6.0540	0.6180	69.3105	4.0356	4.0573	−0.539
313.0	314.0	6.0540	6.7230	0.6690	69.3105	4.0555	4.0511	0.108
314.0	315.0	6.7230	7.3860	0.6630	69.3105	4.0310	4.0449	−0.345
315.0	316.0	7.3860	8.0820	0.6960	69.3105	4.0510	4.0386	0.306
316.0	317.0	8.0820	8.7860	0.7040	69.3105	4.0514	4.0323	0.471
317.0	318.0	8.7860	9.5050	0.7190	69.3105	4.0350	4.0260	0.224
318.0	319.0	9.5050	10.2170	0.7120	69.3105	4.0211	4.0197	0.035
319.0	320.0	10.2170	10.9690	0.7520	69.3105	4.0172	4.0133	0.097
320.0	321.0	10.9690	11.7320	0.7630	69.3105	4.0281	4.0069	0.527
321.0	322.0	11.7320	12.5010	0.7690	69.3104	4.0102	4.0005	0.242
322.0	323.0	12.5010	13.2870	0.7860	69.3104	4.0178	3.9941	0.591
324.0	325.0	14.0830	14.8910	0.8080	69.3104	3.9919	3.9812	0.269
325.0	326.0	14.8910	15.7160	0.8250	69.3104	3.9934	3.9747	0.468
326.0	327.0	15.7160	16.5570	0.8410	69.3104	3.9998	3.9682	0.790
327.0	328.0	16.5570	17.4060	0.8490	69.3104	3.9619	3.9617	0.004
328.0	329.0	17.4060	18.2650	0.8590	69.3104	3.9628	3.9552	0.191
304.0	305.0	1.0030	1.4490	0.4460	69.3106	4.1239	4.1052	0.453
305.0	306.0	1.4490	1.9390	0.4900	69.3106	4.1082	4.0995	0.212
306.0	307.0	1.9390	2.4590	0.5200	69.3106	4.1210	4.0937	0.663
307.0	308.0	2.4590	3.0050	0.5460	69.3106	4.1041	4.0878	0.398
309.0	310.0	3.5720	4.1620	0.5900	69.3106	4.0612	4.0758	−0.359
310.0	311.0	4.1620	4.7780	0.6160	69.3106	4.0717	4.0697	0.050
311.0	312.0	4.7780	5.4000	0.6220	69.3106	4.0711	4.0635	0.186
312.0	313.0	5.4000	6.0510	0.6510	69.3105	4.0764	4.0573	0.467
313.0	314.0	6.0510	6.7090	0.6580	69.3105	4.0458	4.0511	−0.132
314.0	315.0	6.7090	7.3680	0.6590	69.3105	4.0528	4.0449	0.194
315.0	316.0	7.3680	8.0620	0.6940	69.3105	4.0549	4.0387	0.400
316.0	317.0	8.0620	8.7550	0.6930	69.3105	4.0452	4.0324	0.317
317.0	318.0	8.7550	9.4830	0.7280	69.3105	4.0215	4.0260	−0.113
318.0	319.0	9.4830	10.2120	0.7290	69.3105	4.0106	4.0197	−0.227
319.0	320.0	10.2120	10.9540	0.7420	69.3105	4.0343	4.0133	0.520
320.0	321.0	10.9540	11.7140	0.7600	69.3105	4.0259	4.0069	0.471
321.0	322.0	11.7140	12.4790	0.7650	69.3104	3.9974	4.0005	−0.079
322.0	323.0	12.4790	13.2680	0.7890	69.3104	4.0170	3.9941	0.570
323.0	324.0	13.2680	14.0640	0.7960	69.3104	3.9954	3.9877	0.194
324.0	325.0	14.0640	14.8760	0.8120	69.3104	3.9923	3.9812	0.278
325.0	326.0	14.8760	15.7000	0.8240	69.3104	3.9822	3.9747	0.187
326.0	327.0	15.7000	16.5330	0.8330	69.3104	3.9842	3.9683	0.400
327.0	328.0	16.5330	17.3890	0.8560	69.3104	3.9851	3.9618	0.586
329.0	330.0	18.2480	19.1220	0.8740	69.3104	3.9776	3.9488	0.725
304.0	305.0	0.9870	1.4380	0.4510	69.3106	4.1274	4.1053	0.536
305.0	306.0	1.4380	1.9300	0.4920	69.3106	4.1017	4.0995	0.053
307.0	308.0	2.4550	3.0040	0.5490	69.3106	4.0892	4.0878	0.035
308.0	309.0	3.0040	3.5860	0.5820	69.3106	4.0802	4.0818	−0.038
309.0	310.0	3.5860	4.1730	0.5870	69.3106	4.0740	4.0757	−0.042
310.0	311.0	4.1730	4.7790	0.6060	69.3106	4.0721	4.0697	0.060
311.0	312.0	4.7790	5.4030	0.6240	69.3106	4.0755	4.0635	0.294
312.0	313.0	5.4030	6.0440	0.6410	69.3105	4.0799	4.0574	0.552
313.0	314.0	6.0440	6.6960	0.6520	69.3105	4.0594	4.0512	0.203
314.0	315.0	6.6960	7.3630	0.6670	69.3105	4.0498	4.0450	0.120
315.0	316.0	7.3630	8.0630	0.7000	69.3105	4.0565	4.0387	0.440
316.0	317.0	8.0630	8.7530	0.6900	69.3105	4.0452	4.0324	0.317
317.0	318.0	8.7530	9.4840	0.7310	69.3105	4.0248	4.0260	−0.031
318.0	319.0	9.4840	10.2140	0.7300	69.3105	4.0222	4.0197	0.062
319.0	320.0	10.2140	10.9570	0.7430	69.3105	4.0240	4.0133	0.265
320.0	321.0	10.9570	11.7130	0.7560	69.3105	4.0093	4.0069	0.059
321.0	322.0	11.7130	12.4860	0.7730	69.3104	4.0137	4.0005	0.328
322.0	323.0	12.4860	13.2700	0.7840	69.3104	4.0227	3.9941	0.711
323.0	324.0	13.2700	14.0660	0.7960	69.3104	3.9945	3.9877	0.171
324.0	325.0	14.0660	14.8750	0.8090	69.3104	3.9862	3.9812	0.125
325.0	326.0	14.8750	15.7010	0.8260	69.3104	3.9825	3.9747	0.195
327.0	328.0	16.5370	17.3850	0.8480	69.3104	3.9605	3.9618	−0.032
328.0	329.0	17.3850	18.2460	0.8610	69.3104	3.9444	3.9553	−0.276
304.0	305.0	1.0000	1.4490	0.4490	69.3106	4.1384	4.1052	0.802
305.0	306.0	1.4490	1.9420	0.4930	69.3106	4.1188	4.0995	0.469
306.0	307.0	1.9420	2.4640	0.5220	69.3106	4.1187	4.0937	0.608
307.0	308.0	2.4640	3.0100	0.5460	69.3106	4.1002	4.0877	0.304
308.0	309.0	3.0100	3.5770	0.5670	69.3106	4.0950	4.0818	0.323
309.0	310.0	3.5770	4.1630	0.5860	69.3106	4.0895	4.0758	0.336
310.0	311.0	4.1630	4.7760	0.6130	69.3106	4.0890	4.0697	0.473
311.0	312.0	4.7760	5.4200	0.6440	69.3106	4.0734	4.0635	0.243
312.0	313.0	5.4200	6.0610	0.6410	69.3105	4.0577	4.0573	0.009
313.0	314.0	6.0610	6.7120	0.6510	69.3105	4.0454	4.0511	−0.142
314.0	315.0	6.7120	7.3650	0.6530	69.3105	4.0224	4.0450	−0.561
316.0	317.0	8.0670	8.7720	0.7050	69.3105	4.0414	4.0323	0.224
317.0	318.0	8.7720	9.4850	0.7130	69.3105	4.0310	4.0260	0.123
318.0	319.0	9.4850	10.2120	0.7270	69.3105	4.0396	4.0197	0.493
319.0	320.0	10.2120	10.9610	0.7490	69.3105	4.0168	4.0133	0.087
321.0	322.0	11.7180	12.4860	0.7680	69.3104	3.9925	4.0005	−0.201
322.0	323.0	12.4860	13.2740	0.7880	69.3104	4.0119	3.9941	0.444
323.0	324.0	13.2740	14.0690	0.7950	69.3104	3.9863	3.9877	−0.034
324.0	325.0	14.0690	14.8790	0.8100	69.3104	3.9806	3.9812	−0.015
325.0	326.0	14.8790	15.7040	0.8250	69.3104	3.9959	3.9747	0.530
326.0	327.0	15.7040	16.5420	0.8380	69.3104	3.9936	3.9682	0.635
327.0	328.0	16.5420	17.3900	0.8480	69.3104	3.9586	3.9618	−0.080
328.0	329.0	17.3900	18.2530	0.8630	69.3104	3.9525	3.9553	−0.070
323.0	324.0	2.3540	3.1390	0.7850	68.9132	3.9967	4.0144	−0.442
325.0	326.0	3.9350	4.7440	0.8090	68.9131	3.9873	4.0008	−0.339
326.0	327.0	4.7440	5.5660	0.8220	68.9131	4.0043	3.9940	0.257
327.0	328.0	5.5660	6.4000	0.8340	68.9131	3.9799	3.9872	−0.183
328.0	329.0	6.4000	7.2470	0.8470	68.9131	3.9739	3.9804	−0.163
329.0	330.0	7.2470	8.1020	0.8550	68.9131	3.9953	3.9736	0.544
331.0	332.0	8.9740	9.8560	0.8820	68.9131	3.9763	3.9600	0.411
332.0	333.0	9.8560	10.7490	0.8930	68.9130	3.9474	3.9531	−0.145
333.0	334.0	10.7490	11.6520	0.9030	68.9130	3.9317	3.9463	−0.372
334.0	335.0	11.6520	12.5690	0.9170	68.9130	3.9424	3.9395	0.073
335.0	336.0	12.5690	13.4960	0.9270	68.9130	3.9282	3.9327	−0.115
336.0	337.0	13.4960	14.4350	0.9390	68.9130	3.9193	3.9259	−0.169
337.0	338.0	14.4350	15.3820	0.9470	68.9130	3.9059	3.9192	−0.339
338.0	339.0	15.3820	16.3420	0.9600	68.9130	3.9062	3.9124	−0.158
339.0	340.0	16.3420	17.3110	0.9690	68.9130	3.8996	3.9056	−0.154
341.0	342.0	18.2880	19.2790	0.9910	68.9129	3.8973	3.8921	0.133
326.0	327.0	4.7430	5.5650	0.8220	68.9131	3.9846	3.9940	−0.236
327.0	328.0	5.5650	6.3990	0.8340	68.9131	3.9744	3.9872	−0.322
328.0	329.0	6.3990	7.2460	0.8470	68.9131	3.9835	3.9804	0.078
329.0	330.0	7.2460	8.1030	0.8570	68.9131	3.9781	3.9736	0.114
330.0	331.0	8.1030	8.9750	0.8720	68.9131	3.9510	3.9668	−0.399
331.0	332.0	8.9750	9.8560	0.8810	68.9131	3.9488	3.9600	−0.282
333.0	334.0	10.7510	11.6530	0.9020	68.9130	3.9276	3.9463	−0.477
334.0	335.0	11.6530	12.5700	0.9170	68.9130	3.9409	3.9395	0.035
335.0	336.0	12.5700	13.4970	0.9270	68.9130	3.9282	3.9327	−0.115
337.0	338.0	14.4320	15.3830	0.9510	68.9130	3.8941	3.9192	−0.643
338.0	339.0	15.3830	16.3390	0.9560	68.9130	3.9130	3.9124	0.016
339.0	340.0	16.3390	17.3110	0.9720	68.9130	3.9013	3.9056	−0.110
340.0	341.0	17.3110	18.2880	0.9770	68.9130	3.8830	3.8989	−0.408
341.0	342.0	18.2880	19.2780	0.9900	68.9129	3.8904	3.8921	−0.044
326.0	327.0	4.7340	5.5570	0.8230	68.9131	4.0266	3.9940	0.809
328.0	329.0	6.3900	7.2390	0.8490	68.9131	3.9915	3.9804	0.278
329.0	330.0	7.2390	8.0960	0.8570	68.9131	3.9892	3.9736	0.391
330.0	331.0	8.0960	8.9680	0.8720	68.9131	3.9426	3.9668	−0.613
331.0	332.0	8.9680	9.8490	0.8810	68.9131	3.9557	3.9600	−0.108
332.0	333.0	9.8490	10.7420	0.8930	68.9131	3.9565	3.9532	0.084
333.0	334.0	10.7420	11.6480	0.9060	68.9130	3.9613	3.9463	0.377
334.0	335.0	11.6480	12.5660	0.9180	68.9130	3.9286	3.9395	−0.278
335.0	336.0	12.5660	13.4930	0.9270	68.9130	3.9402	3.9327	0.189
336.0	337.0	13.4930	14.4300	0.9370	68.9130	3.9164	3.9259	−0.244
338.0	339.0	15.3810	16.3380	0.9570	68.9130	3.8844	3.9124	−0.720
340.0	341.0	17.3070	18.2850	0.9780	68.9130	3.8838	3.8989	−0.388
344.0	345.0	4.1610	5.1580	0.9970	68.2821	3.8903	3.9052	−0.384
346.0	347.0	6.1670	7.1830	1.0160	68.2821	3.8884	3.8910	−0.068
348.0	349.0	8.2150	9.2530	1.0380	68.2821	3.8908	3.8769	0.357
349.0	350.0	9.2530	10.2990	1.0460	68.2821	3.8877	3.8699	0.458
350.0	351.0	10.2990	11.3590	1.0600	68.2820	3.8768	3.8629	0.359
351.0	352.0	11.3590	12.4220	1.0630	68.2820	3.8547	3.8559	−0.031
352.0	353.0	12.4220	13.4980	1.0760	68.2820	3.8535	3.8490	0.118
353.0	354.0	13.4980	14.5800	1.0820	68.2820	3.8580	3.8420	0.414
355.0	356.0	15.6690	16.7680	1.0990	68.2820	3.8481	3.8283	0.516
356.0	357.0	16.7680	17.8720	1.1040	68.2820	3.8183	3.8214	−0.081
357.0	358.0	17.8720	18.9860	1.1140	68.2820	3.8053	3.8146	−0.244
343.0	344.0	3.1830	4.1660	0.9830	68.2821	3.9207	3.9123	0.213
344.0	345.0	4.1660	5.1600	0.9940	68.2821	3.8980	3.9052	−0.185
345.0	346.0	5.1600	6.1690	1.0090	68.2821	3.9127	3.8981	0.373
346.0	347.0	6.1690	7.1860	1.0170	68.2821	3.8969	3.8910	0.151
347.0	348.0	7.1860	8.2150	1.0290	68.2821	3.8825	3.8840	−0.038
348.0	349.0	8.2150	9.2490	1.0340	68.2821	3.9049	3.8769	0.716
349.0	350.0	9.2490	10.2980	1.0490	68.2821	3.8755	3.8699	0.145
350.0	351.0	10.2980	11.3520	1.0540	68.2820	3.8674	3.8629	0.116
351.0	352.0	11.3520	12.4200	1.0680	68.2820	3.8762	3.8559	0.523
352.0	353.0	12.4200	13.4910	1.0710	68.2820	3.8483	3.8490	−0.017
353.0	354.0	13.4910	14.5750	1.0840	68.2820	3.8484	3.8420	0.165
355.0	356.0	15.6680	16.7650	1.0970	68.2820	3.8405	3.8283	0.319
356.0	357.0	16.7650	17.8710	1.1060	68.2820	3.8116	3.8214	−0.257
357.0	358.0	17.8710	18.9860	1.1150	68.2820	3.8232	3.8146	0.225
346.0	347.0	6.1610	7.1800	1.0190	68.2821	3.9018	3.8910	0.276
347.0	348.0	7.1800	8.2100	1.0300	68.2821	3.8829	3.8840	−0.028
348.0	349.0	8.2100	9.2480	1.0380	68.2821	3.9060	3.8769	0.744
349.0	350.0	9.2480	10.2960	1.0480	68.2821	3.8682	3.8699	−0.044
350.0	351.0	10.2960	11.3530	1.0570	68.2820	3.8858	3.8629	0.589
351.0	352.0	11.3530	12.4200	1.0670	68.2820	3.8622	3.8559	0.163
353.0	354.0	13.4900	14.5730	1.0830	68.2820	3.8594	3.8420	0.450
354.0	355.0	14.5730	15.6650	1.0920	68.2820	3.8591	3.8351	0.621
355.0	356.0	15.6650	16.7630	1.0980	68.2820	3.8387	3.8283	0.272
356.0	357.0	16.7630	17.8690	1.1060	68.2820	3.8265	3.8214	0.133
357.0	358.0	17.8690	18.9840	1.1150	68.2820	3.8220	3.8146	0.194
362.0	363.0	2.6960	3.8200	1.1240	67.4994	3.8018	3.8148	−0.343
364.0	365.0	4.9580	6.1100	1.1520	67.4993	3.8034	3.8007	0.071
365.0	366.0	6.1100	7.2640	1.1540	67.4993	3.7902	3.7937	−0.092
366.0	367.0	7.2640	8.4340	1.1700	67.4993	3.7752	3.7867	−0.305
368.0	369.0	9.6050	10.7850	1.1800	67.4993	3.7638	3.7729	−0.241
370.0	371.0	11.9680	13.1630	1.1950	67.4992	3.7645	3.7592	0.142
371.0	372.0	13.1630	14.3610	1.1980	67.4992	3.7418	3.7524	−0.283
372.0	373.0	14.3610	15.5620	1.2010	67.4992	3.7242	3.7456	−0.575
373.0	374.0	15.5620	16.7710	1.2090	67.4992	3.7163	3.7389	−0.608
374.0	375.0	16.7710	17.9910	1.2200	67.4992	3.7062	3.7322	−0.702
362.0	363.0	2.6960	3.8200	1.1240	67.4994	3.8052	3.8148	−0.253
363.0	364.0	3.8200	4.9580	1.1380	67.4993	3.7887	3.8078	−0.503
364.0	365.0	4.9580	6.1080	1.1500	67.4993	3.7802	3.8007	−0.542
366.0	367.0	7.2660	8.4320	1.1660	67.4993	3.7713	3.7867	−0.409
367.0	368.0	8.4320	9.6030	1.1710	67.4993	3.7539	3.7798	−0.689
368.0	369.0	9.6030	10.7840	1.1810	67.4993	3.7683	3.7729	−0.121
369.0	370.0	10.7840	11.9680	1.1840	67.4993	3.7470	3.7660	−0.507
370.0	371.0	11.9680	13.1600	1.1920	67.4992	3.7477	3.7592	−0.306
371.0	372.0	13.1600	14.3580	1.1980	67.4992	3.7537	3.7524	0.035
372.0	373.0	14.3580	15.5660	1.2080	67.4992	3.7337	3.7456	−0.319
373.0	374.0	15.5660	16.7710	1.2050	67.4992	3.7241	3.7389	−0.398
374.0	375.0	16.7710	17.9900	1.2190	67.4992	3.6977	3.7322	−0.934
362.0	363.0	2.6850	3.8120	1.1270	67.4994	3.8043	3.8148	−0.277
363.0	364.0	3.8120	4.9510	1.1390	67.4994	3.8016	3.8078	−0.162
364.0	365.0	4.9510	6.1020	1.1510	67.4993	3.7799	3.8007	−0.551
366.0	367.0	7.2600	8.4260	1.1660	67.4993	3.7689	3.7867	−0.473
367.0	368.0	8.4260	9.5970	1.1710	67.4993	3.7665	3.7798	−0.353
368.0	369.0	9.5970	10.7770	1.1800	67.4993	3.7753	3.7729	0.064
369.0	370.0	10.7770	11.9620	1.1850	67.4993	3.7303	3.7660	−0.957
371.0	372.0	13.1560	14.3540	1.1980	67.4992	3.7279	3.7524	−0.657
372.0	373.0	14.3540	15.5580	1.2040	67.4992	3.7290	3.7456	−0.446
373.0	374.0	15.5580	16.7670	1.2090	67.4992	3.7244	3.7389	−0.390
374.0	375.0	16.7670	17.9870	1.2200	67.4992	3.7094	3.7322	−0.615
384.0	385.0	5.2340	6.4890	1.2550	66.5677	3.6618	3.6986	−1.005
385.0	386.0	6.4890	7.7490	1.2600	66.5676	3.6715	3.6919	−0.557
386.0	387.0	7.7490	9.0170	1.2680	66.5676	3.6587	3.6853	−0.728
387.0	388.0	9.0170	10.2910	1.2740	66.5676	3.6481	3.6787	−0.840
388.0	389.0	10.2910	11.5690	1.2780	66.5676	3.6596	3.6722	−0.344
389.0	390.0	11.5690	12.8490	1.2800	66.5676	3.6275	3.6657	−1.053
391.0	392.0	14.1330	15.4230	1.2900	66.5676	3.6148	3.6528	−1.052
392.0	393.0	15.4230	16.7150	1.2920	66.5675	3.6268	3.6465	−0.542
393.0	394.0	16.7150	18.0130	1.2980	66.5675	3.6123	3.6401	−0.771
394.0	395.0	18.0130	19.3160	1.3030	66.5675	3.6042	3.6339	−0.823
383.0	384.0	3.9870	5.2350	1.2480	66.5677	3.7024	3.7053	−0.079
384.0	385.0	5.2350	6.4880	1.2530	66.5677	3.6589	3.6986	−1.085
385.0	386.0	6.4880	7.7480	1.2600	66.5676	3.6645	3.6919	−0.749
386.0	387.0	7.7480	9.0160	1.2680	66.5676	3.6332	3.6853	−1.435
387.0	388.0	9.0160	10.2910	1.2750	66.5676	3.6334	3.6787	−1.248
388.0	389.0	10.2910	11.5650	1.2740	66.5676	3.6238	3.6722	−1.336
389.0	390.0	11.5650	12.8480	1.2830	66.5676	3.6245	3.6657	−1.137
390.0	391.0	12.8480	14.1330	1.2850	66.5676	3.6130	3.6593	−1.280
391.0	392.0	14.1330	15.4250	1.2920	66.5676	3.6068	3.6528	−1.276
392.0	393.0	15.4250	16.7150	1.2900	66.5675	3.6203	3.6465	−0.723
394.0	395.0	18.0150	19.3170	1.3020	66.5675	3.5999	3.6339	−0.943
383.0	384.0	3.9890	5.2380	1.2490	66.5677	3.6882	3.7053	−0.464
384.0	385.0	5.2380	6.4930	1.2550	66.5677	3.6722	3.6986	−0.719
386.0	387.0	7.7530	9.0210	1.2680	66.5676	3.6588	3.6853	−0.725
387.0	388.0	9.0210	10.2920	1.2710	66.5676	3.6504	3.6787	−0.776
389.0	390.0	11.5710	12.8530	1.2820	66.5676	3.6378	3.6657	−0.767
390.0	391.0	12.8530	14.1370	1.2840	66.5676	3.6414	3.6592	−0.490
391.0	392.0	14.1370	15.4290	1.2920	66.5675	3.6240	3.6528	−0.796
392.0	393.0	15.4290	16.7200	1.2910	66.5675	3.6287	3.6465	−0.490
393.0	394.0	16.7200	18.0200	1.3000	66.5675	3.6134	3.6401	−0.740
394.0	395.0	18.0200	19.3230	1.3030	66.5675	3.6076	3.6339	−0.728
383.0	384.0	3.9960	5.2430	1.2470	66.5677	3.6990	3.7053	−0.170
384.0	385.0	5.2430	6.5010	1.2580	66.5677	3.6896	3.6986	−0.244
386.0	387.0	7.7620	9.0270	1.2650	66.5676	3.6654	3.6853	−0.543
387.0	388.0	9.0270	10.3030	1.2760	66.5676	3.6604	3.6787	−0.501
388.0	389.0	10.3030	11.5780	1.2750	66.5676	3.6642	3.6722	−0.218
389.0	390.0	11.5780	12.8570	1.2790	66.5676	3.6567	3.6657	−0.246
390.0	391.0	12.8570	14.1420	1.2850	66.5676	3.6623	3.6592	0.084
392.0	393.0	15.4240	16.7180	1.2940	66.5675	3.6453	3.6465	−0.032
393.0	394.0	16.7180	18.0200	1.3020	66.5675	3.6056	3.6401	−0.958
394.0	395.0	18.0200	19.3220	1.3020	66.5675	3.6158	3.6339	−0.499
404.0	405.0	5.5430	6.8680	1.3250	65.5150	3.5958	3.6007	−0.137
405.0	406.0	6.8680	8.1970	1.3290	65.5150	3.5899	3.5945	−0.130
406.0	407.0	8.1970	9.5280	1.3310	65.5150	3.5823	3.5884	−0.170
407.0	408.0	9.5280	10.8640	1.3360	65.5150	3.5661	3.5823	−0.455
408.0	409.0	10.8640	12.1990	1.3350	65.5149	3.5626	3.5763	−0.383
409.0	410.0	12.1990	13.5440	1.3450	65.5149	3.5626	3.5702	−0.214
410.0	411.0	13.5440	14.8900	1.3460	65.5149	3.5595	3.5643	−0.134
411.0	412.0	14.8900	16.2370	1.3470	65.5149	3.5489	3.5583	−0.266
412.0	413.0	16.2370	17.5870	1.3500	65.5149	3.5457	3.5525	−0.191
403.0	404.0	4.2280	5.5470	1.3190	65.5150	3.5929	3.6070	−0.391
404.0	405.0	5.5470	6.8680	1.3210	65.5150	3.6009	3.6007	0.005
405.0	406.0	6.8680	8.1970	1.3290	65.5150	3.6066	3.5945	0.334
406.0	407.0	8.1970	9.5310	1.3340	65.5150	3.5984	3.5884	0.278
407.0	408.0	9.5310	10.8680	1.3370	65.5150	3.5794	3.5823	−0.081
409.0	410.0	12.2040	13.5500	1.3460	65.5149	3.5541	3.5702	−0.454
410.0	411.0	13.5500	14.8930	1.3430	65.5149	3.5339	3.5643	−0.859
413.0	414.0	17.5900	18.9450	1.3550	65.5149	3.5334	3.5466	−0.374
403.0	404.0	4.2220	5.5450	1.3230	65.5150	3.5855	3.6070	−0.599
404.0	405.0	5.5450	6.8680	1.3230	65.5150	3.5884	3.6007	−0.344
405.0	406.0	6.8680	8.1990	1.3310	65.5150	3.5846	3.5945	−0.278
406.0	407.0	8.1990	9.5320	1.3330	65.5150	3.5723	3.5884	−0.451
407.0	408.0	9.5320	10.8720	1.3400	65.5150	3.5776	3.5823	−0.131
408.0	409.0	10.8720	12.2100	1.3380	65.5149	3.5519	3.5762	−0.685
410.0	411.0	13.5550	14.8970	1.3420	65.5149	3.5281	3.5643	−1.025
412.0	413.0	16.2490	17.5970	1.3480	65.5149	3.5189	3.5524	−0.953
413.0	414.0	17.5970	18.9520	1.3550	65.5149	3.5284	3.5466	−0.516
